# Barium and psoriasis: a mini-review and hypothesis linking environmental exposures to ion channel modulation

**DOI:** 10.3389/fmed.2025.1585525

**Published:** 2025-06-18

**Authors:** Jordan Zeldin, Jalin Jordan, Pranav Thota, Richard Vuong, Christeena Jojo, Ian A. Myles

**Affiliations:** ^1^Epithelial Therapeutics Unit, National Institute of Allergy and Infectious Disease, National Institutes of Health, Bethesda, MD, United States; ^2^Stroke, Cognition, and Neuroepidemiology (SCAN) Lab, National Institute of Neurological Disorders and Stroke, National Institutes of Health, Bethesda, MD, United States

**Keywords:** psoriasis, Kv1.3, KCa3.1, barium, inflammation

## Abstract

Psoriasis is a complex autoimmune skin disorder with rising prevalence and significant comorbidities. Although its etiology remains multifactorial, recent epidemiologic and mechanistic studies—including the NHANES-based analysis by Chen et al.—suggest that environmental exposures, particularly to heavy metals may contribute to its pathogenesis. To further understand this association, we reviewed current literature on the pathogenesis of psoriasis and relevant ion channels, as well as the interactions of both with the heavy metal barium. Both epidemiologic and laboratory data highlight a potential link between environmental heavy metal exposure and autoimmune dysregulation in psoriasis. This review offers a novel hypothesis that mechanistically links environmental exposures to psoriasis through ion channel modulation. Further research is warranted to elucidate the precise mechanisms by which barium can influence K^+^ channel function and inflammasome activation, potentially offering new approaches for therapeutic interventions in psoriasis and other autoimmune disorders.

## Introduction

Psoriasis is a chronic autoimmune disease of the skin affecting an estimated 2 to 4% of the world’s population and up to 11% in western European countries such as Norway (1, 2). The prevalence of psoriasis has risen steadily over the last several decades, correlating with increases in industrialization (3). Psoriasis can significantly impair one’s quality of life and lead to the development of unhealthy coping mechanisms such as excessive alcohol consumption (4). Furthermore, psoriasis has strong associations with metabolic syndrome diseases, including obesity, hypertension, hyperlipidemia, and type 2 diabetes mellitus and increased risk of coronary artery disease, stroke, and chronic kidney disease; overall, those with severe psoriasis have nearly twice the mortality rate compared to the general population (5, 6).

Psoriasis classically presents as well-demarcated, erythematous plaques with an overlying scale on the extensor surfaces of the skin, like the knees and elbows. Under the microscope, these plaques exhibit hyperplasia, parakeratosis, tortuous papillary blood vessels, and immune cell infiltrates in the dermis and epidermis, particularly neutrophils in the form of Munro abscesses and CD4+ and CD8+ T cells (7, 8). T cells, particularly T-helper (Th-)17 cells, are central to the disease process, with significantly higher numbers found in active lesions compared to healthy skin, producing excessive amounts of cytokines such as interleukin (IL-)17A, IL-22, and IFN-*γ*, which drive keratinocyte proliferation (9, 10). Healed psoriatic lesions maintain upregulation of disease-related genes and abundance of memory T cells capable of causing disease recurrence (11, 12). Recent data in mice reveal a subset of T cells that continuously produce IL-17 even during time of disease inactivity, which may further reinforce the cycle of chronic inflammation in psoriasis (13, 14).

While the exact pathophysiological mechanism behind psoriasis development remains to be elucidated, understanding the triggers may offer insight. It is known that psoriasis is more common in populations of higher latitudes and Western countries and may be triggered by smoking, infection, alcohol, stress, skin trauma, and air pollutants (15, 16). Recent research has further revealed one’s living environment can contribute to both development and triggers of disease. Long term exposure to air pollutants such as particulate matter less than 2.5 microns in size (PM_2.5_), PM_10_, and nitrogen oxides (NO_X_) increased the risk for developing psoriasis. Despite the evidence linking psoriasis to these select environmental toxins, there is still a dearth of literature that analyzes the effect of other pollutants, such as heavy metals. Barium is the primary heavy metal examined in this paper, building on our previous work that identified an epidemiologic association between barium and psoriasis (17), and because of the relatively extensive literature linking barium to potassium channels compared to other heavy metals. This review aims to summarize a hypothesis linking barium with the pathophysiology of psoriasis and suggest future directions for study.

## Epidemiologic studies linking psoriasis and barium

Two recent epidemiologic studies demonstrated an association between barium and psoriasis, both arriving at this same finding using different datasets and methodologies. The first study cross-referenced geographical data among 532 pollutant compounds released from factories across the United States with aggregated diagnostic data by zip code accounting for 1.2 billion billable visits (81% of all billable visits in the US) across 20,000 zip codes (17). The random forest model showed that estimated nitrogen dioxide concentration and recorded barium release from factories were the most important pollutants among those included in the model. A different method, the penalized regression, ranked carbon monoxide as the most important variable, but this did not account for the non-linearities and interactions that may be captured by a random forest. Moreover, carbon monoxide and nitrogen dioxide may serve as abundant, non-specific markers of high-density urban areas, whereas barium is a more unique pollutant exposure.

The second study was a cross-sectional analysis utilizing data from the National Health and Nutrition Examination Survey (NHANES) which included demographic, clinical, and environmental exposure data, as well as urinary measurements of 11 heavy metals (18). To assess the relationship between metal exposure and psoriasis, the study used multivariable logistic regression models adjusted for potential confounders such as age, sex, BMI, smoking status, and socioeconomic factors. This analysis demonstrated a positive association between psoriasis and several metals, including barium, cesium, antimony, uranium, and cadmium with barium demonstrating the strongest association. In this model, the highest quartile of barium exposure was associated with a 1.794 OR (95% CI 1.189–2.743) of having a diagnosis of psoriasis compared to the lowest quartile. Age-stratified analyses further demonstrated that barium remained the most strongly associated metal in individuals under 60 years of age. For the population older than 60 years, antimony had the strongest OR and when analyzed as a continuous variable, cesium had the highest odds ratio. Although the effect sizes varied by subgroup, the more complex models that account for multiple exposure interactions and non-linearity demonstrated that barium was the exposure most consistently associated with psoriasis. The second most consistent exposure across models was cesium.

Limitations inherent to the NHANES design must be considered. As a cross-sectional survey, NHANES captures exposure and outcome data at a single point in time, which precludes any conclusions about causality or temporality. Additionally, urinary metal levels reflect recent rather than long-term exposure and may be influence by individual metabolic differences. While limitations exist, the consistency of barium association across multiple models supports the need for further longitudinal or mechanistic studies.

## Barium and potassium channels

Barium is a heavy divalent alkaline earth metal found naturally in insoluble minerals like barite and witherite, but can also be found in drinking water, food, and industrial pollution (19). Industry application can vary from drilling muds, glass, plastics, cosmetics, paints, and bricks (20). Acute toxic exposure usually occurs from soluble formulations of barium, typically BaCl_2_ as well as other barium salts. In these soluble forms, the salt can dissociate into the ionic form of barium (Ba^2+^), where it is more physiologically active in the body.

**Figure 1:**
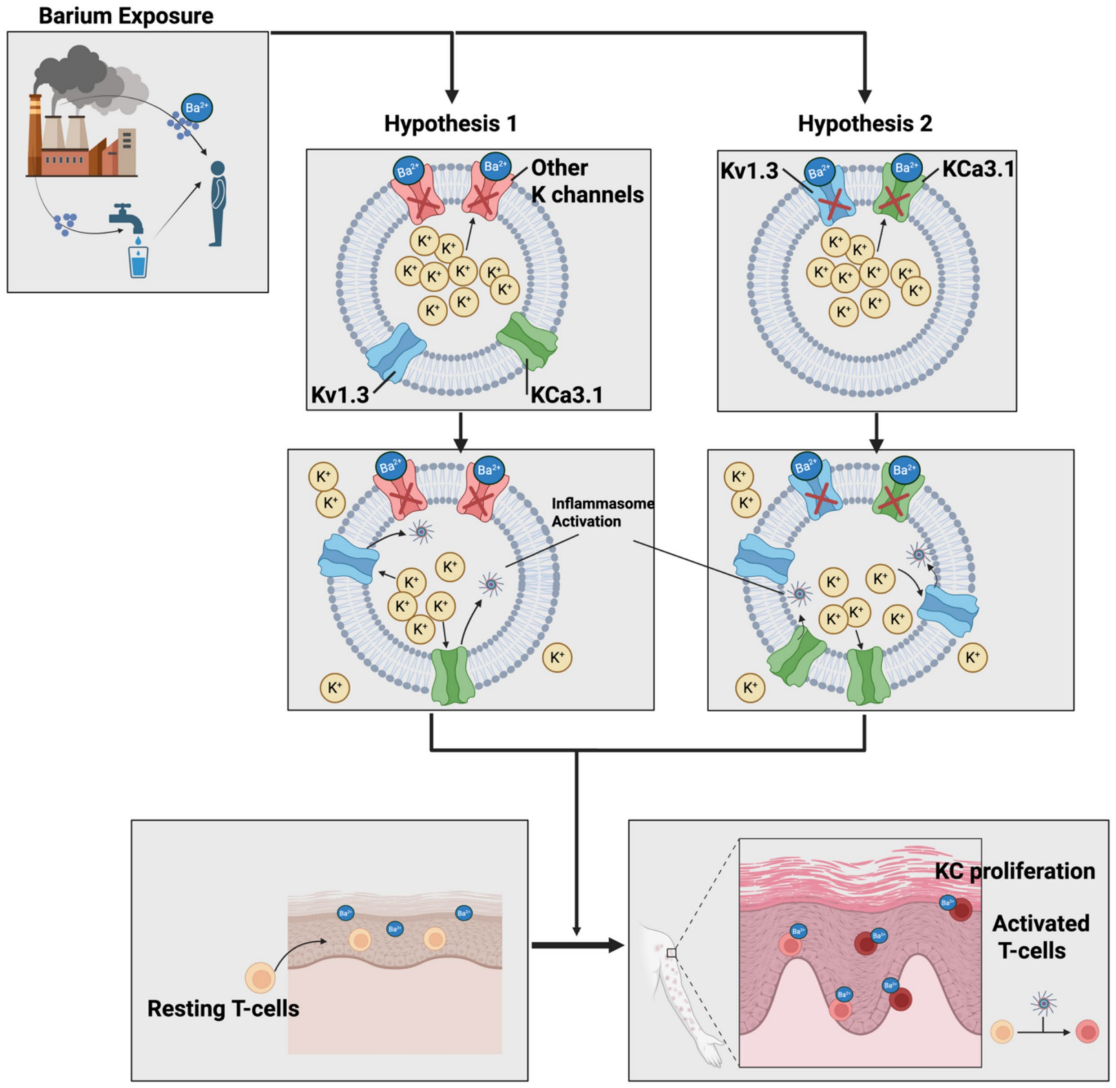
Hypothetical mechanisms of barium driving psoriasis. Chronic exposure to barium from industrial waste leads to the blockage of the potassium channels, preventing K+ efflux. The primary mechanism may entail blockage of non-specific potassium channels (hypothesis 1) or the direct blockage of Kv1.3 and KCa3.1 (hypothesis 2). This blockage may result in either a greater efflux through the Kv1.3 and KCa3.1 channels or greater expression of these channels. Ultimately, both of these effects would cause increased K+ efflux from these proinflammatory channels with a resultant inflammasome activation. When occurring in the T-cells, this would cause activation. KC, keratinocytes.

The primary mechanism of Ba^2+^ in the human body is inhibition of potassium channels. It is hypothesized that because Ba^2+^ has nearly the same ionic radius as K^+^ but with twice the charge, it may bind with higher affinity to the selectivity filter within K^+^ channels (Figure 1); this in turn may allow Ba^2+^ to act as a physical barrier for K^+^ efflux (21, 22). This creates a high intracellular potassium concentration and a low extracellular potassium concentration. Acute barium toxicity is characterized by a profound hypokalemia with sequalae of arrhythmias, muscle weakness, nausea, vomiting, and diarrhea.

In contrast to environmental barium exposure, patients are regularly exposed to barium as a gastrointestinal contrast agent. However, the contrast is formulated as BaSO_4_ (barite), which is insoluble in water and does not readily dissociate into free barium. Insoluble BaSO_4_ may induce toxicity only when enough Ba^2+^ ions are released into the bloodstream, particularly in individuals with colon cancer or local ulcerations or perforation; however, these cases seem to be rare and may not always be due to free barium toxicity but rather complications of barite itself like pulmonary embolism (19, 20). Thus, variable formulations of barium-containing exposures may have differing impacts on potassium metabolism and potassium regulation.

## Potassium channels modulate psoriasis

There are many different types of potassium channels: voltage-gated potassium channels, inward rectifier potassium channels, calcium-activated potassium channels, and two-pore domain potassium channels, each of which may have many different subclasses and be expressed on different types of tissues (23). Barium has been studied on a few of these potassium channels, but not exhaustively (24, 25). Nevertheless, two potassium channels in particular seem likely mechanistic contributors in the link between barium and psoriasis: Kv1.3 and KCa3.1. These are the two most abundantly expressed potassium channels on T-cells isolated from blood, with psoriatic lesions containing increased Kv1.3 expressing T cells compared to normal skin (26, 27). It is possible that chronic barium exposure creates potassium gradients that drive one or both of these channels in the pathogenesis of psoriasis.

### Kv1.3

Kv1.3 is a voltage-gated potassium channel that is abundantly expressed on T-cells and have been implicated in several autoimmune disorders. When an antigen-presenting cell binds to the T-cell receptor, a phosphorylation cascade activates Kv1.3, which cause K + efflux and repolarizing the cell, thus sustaining the electrochemical gradient needed for subsequent calcium influx required for T cell activation (28–31). Recent research has supported a hypothesis that Kv1.3 may contribute to the persistence of chronically stimulated, autoantigen-specific T cells, as evidenced by findings that repeated antigen stimulation biases T cells toward Kv1.3 dependency (26). Psoriasis, type-1 diabetes mellitus, and rheumatoid arthritis have been shown in animal models to exhibit elevated Kv1.3 expression (27, 29).

### KCa3.1

KCa3.1 are calcium-activated potassium channels often expressed in immune cells, epithelial tissue, and endothelial cells—three cell types that are altered in psoriasis. In T-cells, KCa3.1 can compensate for loss of function in Kv1.3 (26). Psoriatic plaques are characterized by hyperkeratosis, which is thought to be related to impaired ability of cells to move calcium intracellularly. Overexpression of KCa3.1 in mice resulted in hyperplasia, hyperkeratosis, and spongiosis, and treatment with a KCa3.1 blocker reversed these histologic changes (32). KCa3.1 channels have also been associated with vascular calcifications, which is more common in psoriasis (33, 34).

## Potassium channels activate the inflammasome

Potassium channels additionally regulate immune responses through the activation of the inflammasome. Inflammasomes are cytosolic protein complexes that are part of the innate immune system. They detect pathogen-associated molecular proteins and damage-associated molecular proteins, which triggers pyroptosis—a form of apoptosis accompanied by the programmed release of inflammatory cytokines, namely IL-1B and IL-18. K^+^ efflux can trigger two types of inflammasomes, NLRP3 and NLRP1. Both inflammasomes have been specifically implicated in psoriasis (35). Psoriasis skin samples had four times the expression of NLRP3 compared to healthy controls. Furthermore, select polymorphisms in NLRP3, as well as the related NRLP1, are associated with increased risk of psoriasis, potentially through increases in IL-1 or IL-18 which are known to be increased in psoriatic lesions (36, 37).

## Other heavy metals of concern

One epidemiologic study found that, second to barium, urinary cesium was associated with a diagnosis of psoriasis, and cesium is also known to inhibit K + channels. Cesium may also bind to K + channels, disrupting K + efflux and leading to hypokalemia (38). This raises the possibility that heavy metals and other pollutants or medications that more broadly interfere with K + channels may induce autoimmune diseases. A few other studies have implicated cadmium, chromium, nickel, lead, and mercury in psoriasis, but these did not test barium (39–41).

## Drug-induced psoriasis and potassium channels

Beyond environmental exposures, certain medications appear able to act on potassium channels and impact the psoriasis susceptibility. The three most common agents implicated in drug-induced psoriasis are beta-blockers, lithium, chloroquine (42). Beta-blockers are known to cause hyperkalemia via inhibition of the Na/K ATPase pump as well as inhibition of renin and thus aldosterone production. Chloroquine inhibits inward rectifying potassium channels in cardiomyocytes (43). Lithium can modify the activity of an inward rectifier in neurons (44). Other agents of drug-induced psoriasis like bupropion and terbinafine have also been found to modify specific potassium channels on particular cell types (45, 46). Insulin is well known to stimulate intracellular potassium shifts, and a case–control study in patients with diabetes suggested a dose-dependent relationship between insulin use and incident psoriasis (47). Lastly, TNF-*α* inhibitors are commonly implicated in drug-induced psoriasis, although through an unclear mechanism. A 2010 study found that prolonged TNF-*α* exposure suppresses the CD3-mediated upregulation of Kv1.3, thus it is possible that inhibition of TNF-α may conversely enhance Kv1.3 expression (48). However, due to wide variety of K channels, heterogenous distribution among tissues, and homeostatic feedback mechanisms for electrolyte regulation, it’s difficult to speculate on a unifying mechanism across different drugs. Thus, further studies demonstrating how these drugs impact Kv3.1 and KCa3.1 in T-cells and epithelial cells would provide further mechanistic insight into the pathology of drug induced psoriasis and relevant inflammatory pathways.

## Therapeutic implications

The role of potassium channels in both environmental and drug-induced psoriasis holds important therapeutic implications, as highlighted in the development of targeted channel blockers for psoriasis and other diseases. Blockers of Kv1.3 have been used therapeutically for psoriasis. Curcumin, a nonselective Kv1.3 blocker, was first revealed in a mouse model to improve psoriasis lesions and lower inflammatory mediators such as TNF-*α*, IFN-*γ*, IL-2, IL-12, IL-22 and IL-23 (49). Later, a meta-analysis of 26 studies using curcumin showed significant improvement in the Psoriasis Area and Severity Index (PASI) score when compared to placebo or when combined with conventional therapy and compared to conventional therapy alone (50). Dalazatide, a selective Kv1.3 blocker, has demonstrated efficacy in reducing the PASI score in psoriasis patients (51). It had also been observed to decrease expression of HLA-DR, Ki67, and CD40L by memory T cells (51). Clinical trials investigating KCa3.1 blockers, such as Senicapoc, in conditions like asthma and sickle cell disease have shown a favorable safety profile but a lack of sufficient clinical efficacy (52). However, their therapeutic potential has not yet been explored in inflammatory skin diseases.

## Discussion

Psoriasis is a complex autoimmune disease primarily mediated by T cells. There is an emerging, albeit partially understood, association suggesting that Kv1.3 may be involved in autoimmunity through alteration in T cell activation. Kv1.3 inhibitors like dalazatide and curcumin have even been used in the treatment of psoriasis. KCa3.1 is also abundantly expressed on T-cells as well as keratinocytes and can mediate T cell activation as well as keratinocyte hyperkeratosis and hyperproliferation. Consistent with this proposed connection: two distinctly designed epidemiologic studies have found an association between barium and psoriasis; barium’s primary mechanism of action in acute toxicity is modulation of K^+^ channels; and several drugs that cause drug-induced psoriasis modulate K^+^ channels.

Together, this raises the intriguing possibility that barium may induce or exacerbate psoriasis by modulating K^+^ homeostasis. However, the exact mechanism is not clear, especially since the effect of barium has not been directly studied on Kv1.3 or KCa3.1. Furthermore, the channels barium has been studies against demonstrate an inhibitory effect of barium. One hypothesis is that barium inhibits other K^+^ channels, creating high intracellular potassium gradients that drive potassium efflux through Kv1.3 and KCa3.1 in T-cells and keratinocytes (Figure 1, hypothesis 1), similar to how digoxin inhibits one channel to drive activity of a different channel. An alternative hypothesis is that chronic exposure, exposure at varying concentrations, or pediatric exposure of barium behaves differently than acute toxicity and that after sustained blockage of Kv1.3 and KCa3.1, there is a compensatory overexpression of the channels (Figure 1, hypothesis 2). Both hypothesis would cause an overall increase in K + efflux through the channels, resulting inflammasome activation and the subsequent psoriasis hallmarks of T-cell signaling and keratinocyte hyperproliferation.

There are multiple limitations to note, first there are limited case reports in the literature on barium toxicity and often lack a clear mechanistic explanation that incorporates cellular ion channel alterations. Next, our epidemiologic studies we used studies are limited in number and do not provide a causal relationship between barium and psoriasis. Further epidemiologic studies investigating the link between barium and psoriasis at the individual level (rather that by zip code) would be useful as well as investigating the link with other autoimmune diseases. In addition, the biggest limitation in a hypothesis connecting barium to psoriasis is the paucity of studies directly linking barium to these particular channels or to T-cell activation. One study, now 25 years old, indicated Ba^2+^ inhibited antigen-specific responses while enhancing PHA-induced activation (53), but further work evaluating the impact of barium on T-cell function is required. While barium has been studied on several K + channels in several tissues, it has not yet been demonstrated how barium might modulate Kv1.3 or KCa3.1 specifically in immune cells or epithelial cells.

In summary, we hypothesize that barium inhibits K^+^ efflux through certain K^+^ channels, creating a high intracellular K^+^ gradient that forces disproportionate K^+^ efflux through these inflammatory K^+^ channels, triggering NLRP3 or NLRP1 signaling, T-cell activation, and keratinocyte hyperproliferation. Future research should utilize longitudinal or cohort-based approaches to definitively establish a link between barium and psoriasis, with findings that may guide regulatory efforts to limit exposure in at-risk populations.
